# Prevalence of Viral Hepatitis B, C, and D in Kazakhstan

**DOI:** 10.1155/2022/9102565

**Published:** 2022-04-22

**Authors:** Almagul Jumabayeva, Alexander Nersesov, Maksut Kulzhanov, Margarita Nefedova, Gulsana Nuraliyeva, Gulnar Rakhimbekova, Shynar Tanabayeva, Ildar Fakhradiyev

**Affiliations:** ^1^S.D. Asfendiyarov Kazakh National Medical University, Almaty, Kazakhstan; ^2^Kazakhstan School of Public Health, Almaty, Kazakhstan; ^3^Algamed Medical Center, Almaty, Kazakhstan; ^4^NJSC “Astana Medical University”, Nur-Sultan, Kazakhstan

## Abstract

**Background:**

Viral hepatitis is a major burden for the healthcare system worldwide. Up to date, a comprehensive analysis of the prevalence of viral hepatitis in Kazakhstan and Central Asia has not been carried out yet. Our epidemiological study aimed at investigating the frequency and spread of viral hepatitis B, C, and D depending on age and sex in Kazakhstan (5-year period).

**Materials and Methods:**

We utilized the data from the primary registration of the incidence of hepatitis B, C, and D in 18 regions of Kazakhstan (period: from 2015 to 2020). Age indicators, gender, and territorial characteristics of registered cases were determined and analysed. The data were obtained from the state information system “Electronic Register of Dispensary Patients”, based on the International Classification of Diseases-10 for coding diseases.

**Results:**

During the period studied, 268 975 cases of hepatitis B, C, and D were detected in Kazakhstan. Hepatitis B was registered in *n* = 109 734 cases. In women, the incidence rate was 40.6% of all cases (*n* = 44545), and in men it was 59.4% (*n* = 65189) of all cases (*p* ≤ 0.01). Hepatitis D was detected in 8 656 cases, of which 58.3% (*n* = 5049) were in men and 41.7% (*n* = 3607) in women (*p* ≤ 0.01). Hepatitis C was registered in *n* = 159 585 cases. The rate was higher in the male population (54.6%; *n* = 82 203) compared to women 45.4% (*n* = 68382) (*p* ≤ 0.01). In 2020 (in comparison with 2015), there was a significant increase in the incidence of hepatitis D by 68.3%, hepatitis B by 49.8%, and hepatitis C by 46.4%. The largest prevalence of hepatitis D was recorded in 2016 which is 22.3% higher compared to 2020. A significant increase in hepatitis C was recorded in 2019 compared to 2015, where indicators were 49.2% higher.

**Conclusion:**

An analysis of the prevalence of hepatitis B, C, and D showed an increase in new cases in Kazakhstan. These findings indicate the need to develop effective preventive measures and screening strategies among people in a high-risk group. The results of the study can be used for the development of a national program to combat the spread of viral hepatitis.

## 1. Introduction

Viral hepatitis is a widespread problem and burden for the healthcare system [1]. It is one of the causes of disability and death in developed and undeveloped countries as a result of liver failure and hepatocellular carcinoma [2]. In 2016, the World Health Organization (WHO) approved the first global health strategy on viral hepatitis with the goal of eliminating viral hepatitis as a major public health threat by 2030 [3].

Despite the difference in the transmission ways of all types of hepatitis [4], the reasons for the continued increase in incidence rates are mainly associated with insufficient attention to health education [5], population growth [6], financial problems, and the low level of general education [7].

The monitoring and surveillance system for viral hepatitis are a priority task of the Ministry of Health of the Republic of Kazakhstan [8]. However, in the context of the public health situation, the Central Asian region has not yet been studied sufficiently [9]. In addition, there is inconsistency in the interpretation of the concepts of “Central” and “Middle” Asia in English-language sources, mainly geographical [10]. It leads to various issues in providing analytical data for review often with an erroneous indication of territorial features.

Previously conducted studies on the analysis of the prevalence of viral hepatitis in Kazakhstan were of a “single-centre” or pilot types [11]. Most of the research was predominantly focused on a narrow sample of subjects, such as donors, HIV-positive patients, etc. [12, 13]. Botheju et al. published a systematic review on the prevalence of hepatitis C in the Middle Asian region [14]. The analysis included studies conducted in Kazakhstan until 2016. The limitations of this work included a narrow study sample, i.e. study was focused on the patients with hepatocellular carcinoma who underwent liver transplantation, drug addicts, HIV-positive patients, etc.

Some studies conducted in the countries of the Middle East, Central, and Middle Asia were mainly focused on the epidemiology of only one type of hepatitis. Hence, such an analysis did not fully reflect the situation in the Central Asian countries due to the lack of a significant database [15]. In addition, considerable differences between the concepts of “Middle Asia” and “Central Asia” in this work were erroneously interpreted as “Central Asia” [15]. In another study, Maistat et al. analysed the epidemiology of only viral hepatitis C in the period 2015–2016 [16]. However, this prevalence analysis was superficial. Moreover, the results of the study were based on the results of a survey that covered the epidemiology of hepatitis C in the general population [16].

In another published review on the prevalence of hepatitis B and C in the Central Asian region, there were no data on population analysis in Kazakhstan [17]. Moreover, the data covered the period only up to 2015. This fact indicates the need to investigate the prevalence of all types of viral hepatitis at the territorial level of the state [18].

This study aimed at the analysis of the prevalence of hepatitis B, C, and D and its relationship with demographic data in Kazakhstan in the period from 2015 to 2020.

## 2. Materials and Methods

### 2.1. Study Design

We used the information from the primary registration database of the incidence of hepatitis B, C, and D in 18 regions of Kazakhstan.

### 2.2. Data Collection

Secondary data from patients of all ages with registered cases of the virus were used for the study. The period of the study was from 2015 to 2020. International Classification of Diseases-10 codes (ICD-10): hepatitis D (B18.0), hepatitis B (B18.1), and hepatitis C (B18.2) were utilized for the study.

Data on the number of dispensary patients were obtained from the Information System of the Republic of Kazakhstan “Electronic Register of Dispensary Patients” (IS-ERDB) [19]. IS-ERDB is designed to form a single centralized information database of patients (electronic register) registered with the dispensary to determine the need for free drug provision at the outpatient level.

IS-ERDB allows employees of medical organizations to automate dispensary registration and observation of patients, storage, and formation of a register of dispensary patients, processing, and provision of statistical and analytical data, including patients with viral hepatitis B, C, and D with acute and chronic forms. The real-time updated IS-ERDB database makes it possible to obtain objective statistical data on the number of patients with viral hepatitis both for individual medical institutions and for all medical organizations of the country.

The study included only initially confirmed cases of viral hepatitis B, C, and D, which were used to determine the age, sex, and territorial characteristics of registered cases.

### 2.3. Antibodies Test

The presence of serological markers of hepatitis B, C, and D virus types was determined using enzyme-linked immunosorbent assays (ELISA) in authorized state laboratories for routine checks [20]. Patient sera were tested for viral markers using seven commercial enzyme immunoassay kits (Dia.Pro Diagnostic BioProbessrl, Milan, Italy) according to the manufacturer's instructions: hepatitis B surface antigen (HBsAg), hepatitis B antigen antibody (HBc Ab), anti -HDV, and anti-HCV.

The licensed test systems were employed ELISA assay. The test system was a set containing microtiter plates necessary for ELISA, reagents, positive and negative samples, the indicators of which are used to calculate the critical optical density, which is a measure of positive and negative results for the samples under study. The quality of test systems was determined by their sensitivity and specificity, according to the instructions for use, the results of determining the sensitivity and specificity of individual series of test systems on reference panels were also taken into account. Samples with inconclusive results (grey area) were retested.

### 2.4. RT-PCR Test

RT-PCR test was conducted according to the recommendation of the Minister of Health of Kazakhstan: order No. 661 (dated by August 23, 2010) “On measures to prevent the incidence of viral hepatitis in the Republic of Kazakhstan” [21]. The study of virus genomes was carried out using highly sensitive quantitative (or qualitative and quantitative) PCR with a lower detection limit of less than 15 IU/ml on analysers with automatic sample preparation in real time and test systems with a high level of analytical reliability [22].

### 2.5. Ethical Issues

Due to the retrospective nature of the study, ethical approval was turned aside by the High Institutional Review Board. The data records were anonymous so that the informed consent was excluded.

### 2.6. Statistical Analysis

Statistical analysis of the results was carried out using SPSS ver. 25.0 (IBM, Armonk, New York, USA). We calculated case-by-case rates (per 100,000 people) with 95% confidence intervals for each individual year and period from 2015 to 2020.

The demographic information was obtained from the Statistics Committee of the Ministry of National Economy of the Republic of Kazakhstan on total population of the country [23]. For time comparison, the method of direct standardization was utilized. Trends in event rate, rates over a specified time interval for men and women were analysed using join point regression, calculating annual percent change (APC), under the assumption of a Poisson distribution. The “*Z*” test was used to find out the difference in proportions.

All results are presented as weighted values. We also used information on the demographic characteristics of the Committee on Statistics of the Ministry of National Economy of the Republic of Kazakhstan for the total population of the Republic of Kazakhstan; *p* < 0.05 was considered statistically significant.

## 3. Results

The age and gender distribution of viral hepatitis B, D, and C for the study period is presented in Table 1. In the period from 2015 to 2020, 268 975 cases of hepatitis B, D, and C were initially detected in Kazakhstan.

Among them, hepatitis D was detected in 8656 cases, of which 58.3% (*n* = 5049) in men (*p* ≤ 0.01) and 41.7% (*n* = 3607) in women. In 98.1% (*n* = 8492) cases, hepatitis D was detected in people over 18 years of age, among which in 58.4% (*n* = 4956) cases it prevailed in men compared to women (41.6%), with a statistically significant difference (*p*=0.02).

The type of hepatitis B was determined in *n* = 109734 cases. In women, it occurred in 40.6% (*n* = 44 545) in men in 59.4% (*n* = 65 189) cases (*p* ≤ 0.01). Hepatitis B prevailed in the adult population (98.1% or *n* = 107 596) in comparison with children under 18 years old (*n* = 2 138). Hepatitis B was more often detected among people over 18 years of age (*p*=0.03): in men in 59.8% (*n* = 64 301) and female population 40.2% (*n* = 43 295).

Hepatitis C was registered in *n* = 159585 cases. It has higher rate in the male population (54.6% or *n* = 82 203) compared to women 45.4% (*n* = 68 382) (*p* ≤ 0.01). Hepatitis C prevailed in the adult population (over 18 years of age) with an indicator of 97.1% (*n* = 146 156) in comparison with youngsters (under the age of 18) (*n* = 4 429). Of all registered cases of hepatitis C among persons over 18 years of age, this type of hepatitis was statistically significantly more common among men in 55% (*n* = 80 443) compared to females (45.0% or *n* = 65 713) (*p*=0.03).

**Figure 1:**
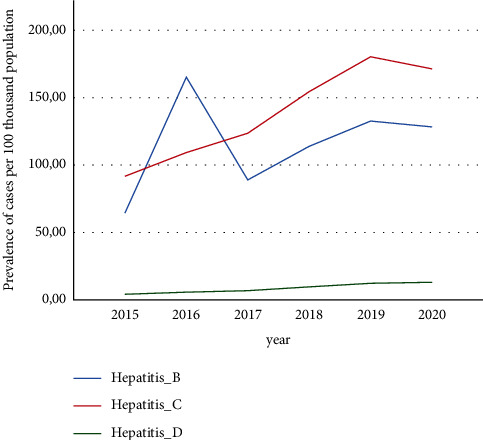
The prevalence of hepatitis B D, and C in the Republic of Kazakhstan per 100 000 people (2015–2020).

The prevalence of hepatitis B, D, and C per 100 000 people in 2015–2020 are presented in Figure 1. Data on hepatitis B showed that in 2015, the prevalence was 64.3 cases per 100 thousand of the population, while in 2016, there was a sharp increase with an indicator of 165.1 cases per 100 thousand of the population. Despite a slight decrease in prevalence rates to 88.97 cases per 100 000 populations in 2016, an increase was observed between 2017 and 2019 with rates of 113.9 cases per 100 000 and 132.6 cases per 100 000 population, respectively. In 2020, the prevalence of hepatitis B was 128.3 cases per 100 000 populations, which is almost twice as high as in 2015 with a statistically significant difference (*p* ≤ 0.01).

Data showed that the prevalence of hepatitis D was low in 2015 (4.13 cases per 100 thousand populations). However, we observed a gradual increase in 2016 up to 5.69 cases per 100 thousand populations, 6.79 cases per 100 000 populations in 2017, and 12.27 cases per 100 thousand. In 2020, the prevalence of hepatitis D was equal to 13.03 cases per 100 thousand (with a statistically significant increase compared to 2015 (*p* ≤ 0.01)).

In 2015, the prevalence of hepatitis C in Kazakhstan was on the level of 91.68 cases per 100 000 populations. In 2016 and 2017, prevalence rate continued to rise up to 123.61 cases per 100 000 and 154.47 cases per 100 000, respectively. In 2019, the highest rate of the prevalence of hepatitis C was recorded, which was equal to 180.35 cases per 100 000 populations. In 2020, the prevalence of hepatitis C was 171.35 cases per 100 000 populations that was statistically significantly higher compared to 2015 (*p* ≤ 0.01).

**Figure 2:**
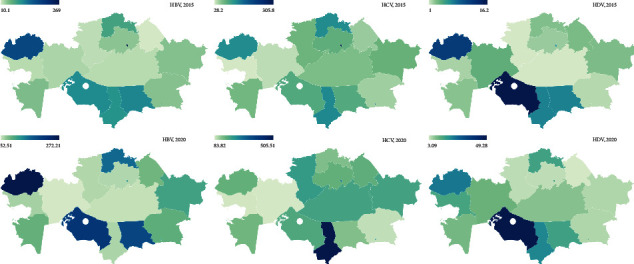
Prevalence of viral hepatitis B, D, and C in 2015 and 2020 on the territory of the Republic of Kazakhstan.

In addition, we analysed the prevalence depending on the region in the territory of Kazakhstan (Figure 2). In 2015, *n* = 720 cases of hepatitis D were registered, of which 97.5% (*n* = 702) were adults and 2.5% (*n* = 18) were children (Appendix 1). The peak prevalence of hepatitis D, equal to 16.2 cases per 100 thousand of the population of Kazakhstan, was registered in the Kyzylorda region, which was several times higher than the national average prevalence (*p* ≤ 0.01). A high prevalence rate (*p* ≤ 0.05), equal to 11.7 cases per 100 thousand population, was found out in the West Kazakhstan region. An average level was observed in Nur-Sultan (6.7) compared to the national average prevalence. The lowest prevalence of hepatitis D (*p* ≤ 0.05), equal to 1.6 and 1.0 cases per 100 thousand population, was in Almaty and Kostanay regions in comparison with the national average prevalence, respectively.

The incidence of hepatitis B in 2015 was on the level of 11 215 cases, among which the proportion of adults and children was 96.6% (*n* = 10 829) and 3.4% (*n* = 386), respectively. The maximum peak prevalence of hepatitis B in 2015 was in Nur-Sultan with an indicator of 269.0 cases per 100 000. It was significantly higher (*p* ≤ 0.01) than the national average prevalence. High prevalence rates were found in a number of regions, such as West Kazakhstan (173.3), Zhambyl (109.6), and Kyzylorda (103.7) cases per 100 thousand. The average prevalence of hepatitis B in 2015 was determined in Almaty (68.5) and Shymkent (56.2) cases per 100 thousand populations. The lowest prevalence of hepatitis B was noted in Atyrau (*p* ≤ 0.01) (19.6) and Pavlodar regions (*p* ≤ 0.01) (10.1) cases per 100 thousand population of Kazakhstan than the national average prevalence.

In 2015, *n* = 15 968 primary cases of viral hepatitis C were registered, of which *n* = 15 318 (96.0%) were adults and *n* = 650 (4.0%) were children. In the country as a whole, in 2015, the peak prevalence of hepatitis C, as well as hepatitis B, was established in Nur-Sultan city (*p* ≤ 0.01). It was equal to 305.8 cases per 100 000, which also exceeded several times the national average prevalence in the country (91.68). The high prevalence of hepatitis C (*p* ≤ 0.05) was determined in the Almaty city, which amounted to 170.3 cases per 100 thousand populations. This number was as almost on the same level as in the West Kazakhstan region (129.4) and the North Kazakhstan region (129.6) cases per 100 000.

Analysis of the data obtained after 5 years (Appendix 2) showed that in 2020, *n* = 2428 primary cases of viral hepatitis D were registered, of which *n* = 2389 (98.3%) were of the adult population and *n* = 39 were children (1 .7%). In 2020, the peak prevalence of hepatitis D was registered in the Kyzylorda region (*p* ≤ 0.01) and equalled 49.3 cases per 100 000, and this figure exceeded the prevalence rate in the country (13.03).

High prevalence rates of hepatitis D were detected in Shymkent city 18.2 cases per 100 thousand population (*p* ≤ 0.01), as well as in the West Kazakhstan region 22.8 cases per 100 thousand population (*p* ≤ 0.01), and in the Turkestan region 20.8 cases per 100 thousand (*p* ≤ 0.01). A low prevalence of hepatitis D in 2020 was observed in Akmola and Pavlodar regions with a level of 4.3 cases per 100 thousand (*p* ≤ 0.01) and 3.1 cases per 100 thousand (*p* ≤ 0.01) compared to the national average prevalence, respectively.

In 2020, *n* = 23 906 cases of hepatitis B were registered, of which 98.6% (*n* = 23 583) were adults and 1.4% (*n* = 323) were children. In terms of the prevalence of hepatitis B in 2020, it can be noted that the peak incidence was determined in West Kazakhstan (272.2) cases per 100 thousand (*p* ≤ 0.01), Kyzylorda region (214.42) cases per 100 thousand people (*p* ≤ 0.01), and in the city of Nur-Sultan (269.6) cases per 100 thousand than the national average prevalence.

A high prevalence of hepatitis B was registered in the Zhambyl region (191.4 cases per 100 thousand) and in the North Kazakhstan region (157.43 cases per 100 thousand). The high level was also observed in the city of Shymkent with an indicator of 183.1 cases per 100 000 people. The low prevalence of hepatitis B was typical for Aktobe (*p* ≤ 0.05) and Karaganda regions (*p* ≤ 0.05), equal to 52.5 cases per 100 000 and 56.9 cases per 100 000 compared to the national average prevalence, respectively.

In 2020, *n* = 31 927 cases of viral hepatitis C were detected, of which *n* = 31 189 (97.7%) were adults and *n* = 738 (2.3%) were children. In 2020, compared to the national average prevalence the peak incidence of hepatitis C was typical for two large cities, such as Nur-Sultan (267.1) cases per 100 000 populations (*p* ≤ 0.01) and Shymkent (240.4) cases per 100 000 populations (*p* ≤ 0.01), as well as the Turkestan region (265.1) cases per 100 thousand populations.

High prevalence rates of hepatitis C in 2020 were typical for Almaty city (222.1 cases per 100 thousand population), as well as Karaganda and Kostanay regions with rates equal to 192.2 and 206.8 cases per 100 thousand populations, respectively. In 2020, in the Almaty region (*p* ≤ 0.05), a low prevalence of hepatitis C was determined that was equal to 94.8 cases per 100 thousand of the population. The low incident rates were also recorded in the Aktobe region (*p* ≤ 0.01) (83.2) cases per 100 thousand and Atyrau region (*p* ≤ 0.01) (85.1) cases per 100,000 populations.

Analysis of the obtained data showed that in 2020 compared to 2015, there was a significant increase in hepatitis D by 68.3%, hepatitis B by 49.8%, and hepatitis C by 46.4% (*p* ≤ 0.01). In 2016, a high increase in delta hepatitis was recorded, which was 22.3% higher compared to 2020 (*p* ≤ 0.05). The largest increase in hepatitis C was recorded in 2019, compared to 2015, the figures were higher by 49.2% (*p* ≤ 0.01) compared to the national average prevalence.

## 4. Discussion

Despite significant progress in prevention and treatment, viral hepatitis continues to be of great burden for the healthcare system in Central Asia and Kazakhstan. To the best of our knowledge, this is the first study to analyse the prevalence of viral hepatitis B, D, and C in Kazakhstan over the past 5 years.

In the period from 2015 to 2020, 268 975 cases of hepatitis B, D, and C were initially detected in Kazakhstan. According to our results, the prevalence of viral hepatitis among males (*p* ≤ 0.01) was observed, including a population over 18 years of age. The epidemiology of viral hepatitis demonstrates gender differences, for example, the male gender has been associated with accelerated liver fibrosis [24], while women have relatively low rates of liver cirrhosis [25].

In fact, the global prevalence of hepatitis B varies greatly. There are high (>8%), intermediate (2–7%), and low (<2%) endemic areas [26].

In fact, hepatitis B prevalence varies geographically: 6% in Africa, 2% in Southeast Asia, and 1% in the America [27]. According to the National Health and Nutrition Examination Survey (NHANES), the prevalence of chronic hepatitis B virus infection in the United States is 1.59 million (range 1.25–2.49 million) [28]. In the countries of the European Union in the period 2005–2015, there was a relatively low prevalence of viral hepatitis B and C. Since, based on estimates of the population and donated blood, the overall prevalence of hepatitis B in these countries is estimated at 0.9% (95% CI 0.7–1.2), which corresponds to almost 4 .7 million HBsAg-positive cases and an overall HCV prevalence of 1.1% (95% CI 0.9–1.4), representing 5.6 million anti-HBV-positive cases [29]. In the context of the epidemiological situation in China, the incidence of viral hepatitis, due to good three-dose vaccination coverage over the past decade, indicates good control of the situation. However, the situation with hepatitis C shows more disappointing figures, according to which in some provinces the incidence is 10/100 thousand of the population [30].

Previous studies of the prevalence of viral hepatitis in Central Asian countries showed a decrease in the prevalence of viral hepatitis B in Kazakhstan [31]. We believe that this was due to the study of the prevalence of viral hepatitis in a specific cohort and not in the entire population of the country. The same significant difference is noted in another study, where the prevalence of hepatitis B and C in 2005 was 10% and 3.1%, respectively [32].

Our data indicate the absence of accurate domestic studies of the prevalence of hepatitis B and C among the population of the Republic of Kazakhstan [33].

### 4.1. Hepatitis B

According to our data, the peak prevalence of hepatitis B in 2015 in Nur-Sultan city was also identified in 2020. In addition, high prevalence rates in 2015 in West Kazakhstan and Kyzylorda regions were replaced by a peak increase in new cases in 2020. Moreover, a five-year analysis of cases showed that in 2016, there was an outbreak of viral hepatitis B.

Given the predominantly transfusion route of transmission of viral hepatitis B, transmission of hepatitis through contaminated donated blood may also affect its prevalence. According to the results of a study conducted in Nur-Sultan among blood donors, the estimated risk of transfusion infection was as follows: HIV-1.2, hepatitis B-125.4, and hepatitis C-137.7 (prevalence per 1 million donors) [34]. In addition, according to the results of a number of studies, the defective inadequacy of donor blood (by 2016) increased mainly due to an increase in the prevalence of transfusion-transmissible infections by 47.6% [35]. At present, the nucleic amplification technique (NAT) has been used with sufficiently high efficiency for screening donated blood in Kazakhstan [36]. However, a growing body of evidence suggests that there may still be limited risk of hepatitis transmission from donors with occult viral hepatitis with extremely low viral loads that are not detectable by ID-NAT [37].

### 4.2. Hepatitis D

The peak increase in cases of viral hepatitis D remained in the Kyzylorda region both in 2015 and 2020. In 2020, we observed the high increase in hepatitis D in the city of Shymkent and region.

Unlike hepatitis B and C, the growth of hepatitis D in the period 2015–2020 had a systematic character, without sharp jumps and fluctuations. However, unlike hepatitis B and C, the incidence of hepatitis D continued to increase in 2020.

According to our results, hepatitis D showed a growth of 63.8% over 5 years. It is well known that viral hepatitis causes about 1.34 million deaths annually, of which 66% of deaths are due to the incidence of viral hepatitis B [38]. Moreover, the incidence of hepatitis D is usually associated with the most severe forms of acute and chronic viral hepatitis [39]. It has been estimated that about 5% of HbsAg carriers worldwide are coinfected with hepatitis D [39, 40]. According to Miao et al., there are between 48 and 60 million HDV infections among people infected with HBV, resulting in a global prevalence of 0.80% in the general population and 13.02% in HBsAg-positive carriers [39]. Given this fact, it is necessary to recall the importance of monitoring proper immunization against hepatitis B in order to prevent the development of hepatitis D [41].

### 4.3. Hepatitis C

In 2020, in addition to the city of Nur-Sultan (with a peak incidence in 2015), Shymkent and the Turkestan region also showed peak growth. 2015 is characterized by a high increase in new cases of hepatitis C for the city of Almaty, West Kazakhstan and North Kazakhstan regions. For 2020, a high prevalence of hepatitis C has also been identified in the cities of Almaty, Karaganda, and Kostanay. It should also be noted that an outbreak of new cases of hepatitis C was detected in 2019.

Since hepatitis B and C are mostly asymptomatic, many cases were undiagnosed and untreated [42]. The high increase in the incidence of hepatitis in the Republic of Kazakhstan over a 5-year period, despite the widespread use of vaccination among the population, may be associated with the improvement of diagnostic measures. In fact, blood donors, pregnant women, military personnel, and patients with indications for surgery are subject to mandatory examination for the detection of markers of viral hepatitis B and C. In addition, the increase in the number of dental clinics, as indicated in earlier studies, due to not the imperfect sterilization can also contribute to an increase in infection with viral hepatitis B and C [43]. Therefore, these facts show the importance of careful blood screening and other procedural checks [44].

Over the five-year period under study, the prevalence of hepatitis C showed a consistently high growth. However, in 2020, there was a slight decrease in the incidence of hepatitis C, similar to hepatitis B.

As previously mentioned, WHO is implementing global viral hepatitis elimination programs by 2030 worldwide, which aims to reduce new infections by up to 90% and deaths from viral hepatitis by 65% [45].

These viral hepatitis control programs cover a number of sectors such as testing, treatment, hepatitis B virus immunization, prevention of mother-to-child transmission, and reduction of the risk of blood transmission [46].

However, due to the concentration of health forces on the fight against the COVID-19 pandemic, the diagnosis and treatment of chronic diseases have suffered [47]. A slight decrease in the prevalence of hepatitis B and C could be due to the difficult epidemiological situation with COVID-19 observed in Kazakhstan last year.

According to the results obtained (presented on the prevalence map), one can note the high prevalence of viral hepatitis in the southern regions of Kazakhstan for the entire period of the study (Figure 2). This fact is most likely associated with the relatively low level of well-being of the inhabitants of the southern regions. In fact, the level of poverty in the southern regions of the country exceeds this indicator than in other regions of the Republic of Kazakhstan [48]. In addition, it should be noted that the number of residents living in rural areas also prevails in the southern regions [49]. This may be due to the low level of health education in rural areas [50].

Despite the increase in cases of detection of viral hepatitis relative to the country's population in percentage terms, in Kazakhstan, compared with other countries, the prevalence is below 0.2% for hepatitis B and C and 0.01% for hepatitis D.

At present, control measures, such as vaccination, risk reduction, and health education, remain important and effective to combat the continued rise in viral hepatitis [51].

In the case of hepatitis B, infection detection and, in some cases, lifelong therapy will continue to play an important role in its elimination [52]. In the case of hepatitis C, the existing therapy has proven to be highly effective and with a low number of side effects [53]. However, only a strategy aimed at increasing the testing of the population, especially high-risk groups (drug users, sex workers, prisoners, and homeless people) [54–56], can reduce the prevalence of viral hepatitis in Kazakhstan.

### 4.4. Study Limitations

This study has several limitations. The study did not analyse the prevalence of viral hepatitis depending on the age breakdown of patients according to the WHO age classification. It makes it impossible to accurately determine the reasons for the increase in registrations during the study period.

## 5. Conclusion

An analysis of the prevalence of hepatitis B, C, and D showed a high increase in new cases in Kazakhstan in the period 2015–2020. Our findings indicate that viral hepatitis remains problem for public health in Kazakhstan and the Central Asian region. This fact demonstrates the need to develop effective preventive measures, including improving the health education of the population and the availability of diagnostics. In this regard, a strategy aimed at increasing the testing of the population and especially certain individuals at a high-risk group for infection with viral hepatitis can play a vital role in the reduction of the prevalence of viral hepatitis in Kazakhstan. The results of the study can be used to improve and optimize the state program to combat all types of hepatitis in the region.

## Figures and Tables

**Table 1:** Distribution of viral hepatitis B, D, and C by age and sex.

Age	B18.0 (В + D) (n, (%))			*p*	B18.1 (В without D) (n, (%))			*p*	B18.2 (С) (n, (%))			*p*
	Female	Male	Total		Female	Male	Total		Female	Male	Total	
Younger than 18	71 (2.0)	93 (1.8)	164 (1.9)	0.12	888 (1.4)	1.250 (2.8)	2.138 (1.9)	0.24	1.760 (2.2)	2.669 (3.9)	4.429 (2.9)	0.04
Older than 18	3.536 (98.0)	4.956 (98.2)	8.492 (98.1)	0.02^*∗*^	64.301 (98.6)	43.295 (97.2)	107.596 (98.1)	0.03^*∗*^	80.443 (97.8)	65.713 (96.1)	146.156 (97.1)	0.03^*∗*^
Total	3607 (41.7)	5049 (58.3)	8656 (100)	0.01^*∗*^	65.189 (59.4)	44.545 (40.6)	109.734 (100)	0.01^*∗*^	82.203 (54.6)	68.382 (45.4)	150.585 (100)	0.01^*∗*^

^*∗*^ *Z*-test, *p* ≤ 0.05.

## Data Availability

All available data were included in the text of the manuscript.

## Supplementary Materials

Supplementary Materials Appendix 1: the prevalence of viral hepatitis B, D, and C for 2015 by regions of the Republic of Kazakhstan. Appendix 2: the prevalence of viral hepatitis B, D, and C for 2020 by regions of the Republic of Kazakhstan.
