# Supplementary material for: Prevalence of Viral Hepatitis B, C, and D in Kazakhstan
Source: ScientificWorldJournal. 2022 Apr 22;2022:9102565. doi: 10.1155/2022/9102565 (PMC9054462; doi:10.1155/2022/9102565)
Supplement: Supplementary Materials — Appendix 1: the prevalence of viral hepatitis B, D, and C for 2015 by regions of the Republic of Kazakhstan. Appendix 2: the prevalence of viral hepatitis B, D, and C for 2020 by regions of the Republic of Kazakhstan. [file 9102565.f1.zip › 9102565.f1/Appendix 1.docx]

Appendix 1: The prevalence of viral hepatitis B, D and C for 2015 by regions of the Republic of Kazakhstan.

| **Region** | **Prevalence of cases per 100 000 population** | | |
| --- | --- | --- | --- |
|  | **B18.0 (В + D)** | **B18.1 (В without D)** | **B18.2 (С)** |
| Akmola region | 2,2 | 35,4 | 80,2 |
| Aktobe region | 3,8 | 24,6 | 36,6 |
| Alma-Ata's region | 1,60 | 38,3 | 47,7 |
| Atyrau region | 1,9 | 19,6 | 28,2 |
| West-Kazakhstan region | 11,7 | 173,3 | 129,4 |
| Jambyl Region | 7,0 | 109,6 | 88,2 |
| Karaganda region | 3,0 | 19,7 | 63,6 |
| Kostanay region | 1,0 | 17,6 | 72,0 |
| Kyzylorda Region | 16,2 | 103,7 | 90,2 |
| Mangistau region | 2,6 | 42,0 | 69,7 |
| South Kazakhstan region | 2,4 | 40,6 | 41,2 |
| Pavlodar region | 2,1 | 10,10 | 50,0 |
| North-Kazakhstan region | 2,8 | 76,1 | 129,6 |
| The East Kazakhstan region | 2,9 | 39,2 | 76,4 |
| Nur-Sultan | 6,7 | 269,0 | 305,8 |
| Almaty city | 3,0 | 68,5 | 170,3 |
| Shymkent | 4,6 | 56,2 | 90,3 |
