# Supplementary material for: Prevalence of Viral Hepatitis B, C, and D in Kazakhstan
Source: ScientificWorldJournal. 2022 Apr 22;2022:9102565. doi: 10.1155/2022/9102565 (PMC9054462; doi:10.1155/2022/9102565)
Supplement: Supplementary Materials — Appendix 1: the prevalence of viral hepatitis B, D, and C for 2015 by regions of the Republic of Kazakhstan. Appendix 2: the prevalence of viral hepatitis B, D, and C for 2020 by regions of the Republic of Kazakhstan. [file 9102565.f1.zip › 9102565.f1/Appendix 2.docx]

Appendix 2: The prevalence of viral hepatitis B, D and C for 2020 by regions of the Republic of Kazakhstan.

| **Region** | **Prevalence of cases per 100 000 population** | | |
| --- | --- | --- | --- |
|  | **B18.0 (В + D)** | **B18.1 (В without D)** | **B18.2 (С)** |
| Akmola region | 4.3 | 62,6 | 160,5 |
| Aktobe region | 10,8 | 52,5 | 83,8 |
| Alma-Ata's region | 5.3 | 94,1 | 94,8 |
| Atyrau region | 14,7 | 66,5 | 85,1 |
| West-Kazakhstan region | 22,8 | 272,2 | 159,5 |
| Jambyl Region | 15,6 | 191,4 | 133,1 |
| Karaganda region | 8.2 | 56,9 | 192,2 |
| Kostanay region | 4,5 | 62,9 | 206,8 |
| Kyzylorda Region | 49,3 | 214,4 | 179,4 |
| Mangistau region | 9,7 | 91,3 | 148,8 |
| South Kazakhstan region | 20,8 | 64,6 | 265,1 |
| Pavlodar region | 3.1 | 86,9 | 162,0 |
| North-Kazakhstan region | 15,3 | 157,4 | 135,3 |
| The East Kazakhstan region | 5.3 | 110,2 | 192,1 |
| Nur-Sultan | 15,7 | 269,6 | 267,1 |
| Almaty city | 12.1 | 123,1 | 222,1 |
| Shymkent | 18.2 | 183,1 | 240,4 |
